# Area deprivation and age related macular degeneration in the EPIC-Norfolk Eye Study

**DOI:** 10.1016/j.puhe.2014.10.012

**Published:** 2015-02

**Authors:** Jennifer L.Y. Yip, Anthony P. Khawaja, Michelle P.Y. Chan, David C. Broadway, Tunde Peto, Robert Luben, Shabina Hayat, Amit Bhaniani, Nick Wareham, Paul J. Foster, Kay-Tee Khaw

**Affiliations:** a Department of Public Health & Primary Care, University of Cambridge, Cambridge, UK; b NIHR Biomedical Research Centre at Moorfields Eye Hospital and UCL Institute of Ophthalmology, London, UK; c Department of Ophthalmology, Norfolk and Norwich University Hospital, Norwich, UK; d MRC Epidemiology Unit, University of Cambridge School of Clinical Medicine, Cambridge, UK

**Keywords:** Area deprivation, Inequalities, Macular degeneration

## Abstract

**Objectives:**

To investigate the relationship between area deprivation, individual socio-economic status (SES) and age related macular degeneration (AMD).

**Study design:**

Cross sectional study nested within a longitudinal cohort study.

**Methods:**

Data were collected in the EPIC-Norfolk Eye Study by trained nurses, using standardized protocols and lifestyle questionnaires. The English Index of multiple deprivation 2010 (IMD) was derived from participants' postcodes. AMD was identified from standardized grading of fundus photographs. Logistic regression was used to examine associations between IMD, SES and AMD.

**Results:**

5344 pairs (62.0% of total 8623) of fundus photographs were of sufficient quality for grading of AMD. Of 5182 participants with complete data, AMD was identified in 653 participants (12.60%, 95%CI = 11.7–13.5%). Multivariable logistic regression showed that people living in the most affluent 5% of areas had nearly half the odds of AMD compared to those living in comparatively more deprived areas (OR = 0.56, 95% CI = 0.36–0.89, *P* = 0.02), after adjusting for age, sex, education, social class and smoking.

**Conclusions:**

The authors found that living in the most affluent areas exerted a protective effect on AMD, independently of education and social class. Further investigation into underlying mechanisms will inform potential interventions to reduce health inequalities relating to AMD.

## Highlights

• The relationship between area deprivation and AMD diagnosed from fundus photographs was examined.
• Data from 5182 participants from the EPIC-Norfolk Eye study were analyzed.
• Predominantly early AMD was identified in 653 participants (12.6%).
• People living in more affluent areas had nearly half the risk of AMD.
• Results require interpretation with caution due to the healthy population studied.

## Introduction

Age-related macular degeneration (AMD) is a leading cause of low vision and blindness in developed countries.^1^ Early disease with minor symptoms can progress to either geographic atrophy (‘dry’ AMD) or choroidal neovascularisation (‘wet’ AMD), both of which can have devastating effects on central vision in late stages of the condition. AMD causes approximately 5% of global blindness, with an estimated 71,000 new cases of late AMD per year in the UK.^2^ Furthermore, both incidence and prevalence are expected to increase due to the ageing population structure since older age is the strongest risk factor for AMD. Pooled findings from three continents showed that the prevalence of AMD was 0.2% in those aged 55–64 years compared to 13.1% in people aged 85 and over.^3^

Rudolf Virchow was one of the first physicians to identify medicine as a social science.^4^ Since then, health inequalities and the importance of social causes of poor health have been highlighted in public health policy by the Black report,^5^ the Acheson report^6^ and the WHO Commission on Social Determinants of Health.^7^ People living in poorer areas, from less affluent backgrounds, have a higher risk of morbidity and mortality. There is substantial evidence that lower socio-economic status (SES) is associated with visual impairment (VI),^8–10^ higher prevalence and incidence of eye disease^11,12^ and ocular risk factors.^13–16^ However, less is known about the relationship between eye health and area deprivation. The impact of individual SES on health can differ from the effects of the local social and physical environment. Studies investigating the relationship between area deprivation and adverse visual outcomes have provided mixed results. Neighbourhood deprivation has been linked to late presentation of glaucoma,^15,16^ acute angle closure incidence^17^ and variations in provision of eyecare services in the UK.^18,19^ Fraser and co-workers found that people living in more deprived areas were more likely to present in the late stages of glaucoma and that this effect was partly accounted for by optometry access.^16^ However, studies from Australia^20^ and the UK^21,22^ have failed to demonstrate an association between area deprivation and VI. Deprivation was not associated with visual acuity at presentation in a study of 240 hospital records of patients with exudative AMD from two Scottish National Health Trusts. A recent study of routine data has shown that certification of visual impairment was not associated with deprivation^21^; although the authors suggested that variations in the registration process may have contributed to their conclusions.

The authors have previously reported that area deprivation was associated with a higher prevalence of low vision, and that this effect was independent of individual SES. There is a potential association between AMD and area deprivation through mutual risk factors such as smoking and poor diet or poorer access to health care. In the present study, it was sought to understand the relationship between deprivation and eye health further by examining the relationship between area deprivation, individual SES and AMD.

## Methods

The European Prospective Investigation into Cancer and Nutrition study (EPIC) is a 10 country collaborative cohort study investigating lifestyle and nutritional risk factors for cancer. Detailed descriptions of the study methods and recruitment have been reported previously.^23,24^ Data for the present study, the EPIC-Norfolk Eye Study was collected between 2004 and 2011, and was based on the third round of clinical examinations, including the pilot phase between 2004–2006.^24^ All participants completed a detailed self-administered health and lifestyle questionnaire and attended a local clinic for a physical examination.

Monocular visual acuity (VA) was measured using a LogMAR chart (Precision Vision, LaSalle, IL, USA) with the aid of the participant's usual distance correction at 4 metres (m) (or 2m then 1m if unable to read any letters). Educational attainment, socio-economic status, demographic and lifestyle characteristics were ascertained using the health questionnaire. Educational attainment was recorded and classified into four groups (less than O-level, up to and including O-level, up to and including A-level, university degree or postgraduate qualifications) according to the highest qualification achieved. Social class was recorded using the Registrar General's occupation based classification system: social class I are professionals, class II include managerial and technical occupations, class III is sub-divided into non-manual (Class IIInm) and manual skilled (Class IIIm) workers, class IV consists of partly skilled workers and class V are unskilled manual workers. For the purposes of this study, social class was dichotomized into non-manual (Class I-IIInm) and manual (Class IIIm-V). Smoking status was recorded as current, former or never and alcohol consumption was recorded as units consumed in the past week, using standard photographs to guide estimates of one unit. Deprivation indices for each participant were derived from data linkage of postcodes of the participant's reported permanent address at the time of the eye examination.

Usual physical activity was assessed using two questions relating to activity in the past year. The first question asked about physical activity at work, classified into four categories: sedentary, standing (e.g. hairdresser or guard), physical work (e.g. plumber, nurse) and heavy manual work (e.g. construction worker). The second question asked about hours per week spent in other physical activity for both winter and summer (recreational activity). A simple index allocated each individual into four categories: inactive (sedentary job with no recreational activity); moderately inactive (sedentary job with <0.5h recreational activity per day or standing job with no recreational activity); moderately active (sedentary job with 0.5–1h recreational activity or standing job with <0.5h recreational activity per day or physical job with no recreational activity); and active (sedentary job with >1h recreational activity per day or standing job with >1h recreational activity per day or physical job with at least some recreational activity per day or heavy manual job). The physical activity scale used has been validated against heart rate monitoring with individual calibration in independent studies.^25,26^ In addition, the index is also inversely related to all cause mortality and incidence of cardiovascular disease in the EPIC-Norfolk cohort.^27^

The index of multiple deprivation (IMD) has been used frequently as a measure of relative deprivation to guide resource allocation and provision of services in the UK.^28^ Deprivation in this context refers to the relative disadvantage an individual experiences living in a certain neighbourhood. The IMD is based on 38 routinely collected indicators, aggregated into seven weighted domains to represent different dimensions of deprivation, namely income, employment, health and disability, education and skills, barriers to housing and services, crime and environment. The aggregate IMD is generated for each local super output area (LSOA) in England. Each LSOA is delineated using data from the 2001 census and has a minimum of 1000 residents, 400 households and an average population of 1500 residents, correlating to a socially homogenous area. The theoretical basis, validity and reliability of IMD has been widely discussed.^28^

### Ascertainment of AMD

Digital fundus photographs of the optic disc and macula were taken using a TRC-NW6S non-mydriatic retinal camera and IMAGEnet Telemedicine System (Topcon Corporation, Tokyo, Japan) with a 10 megapixel Nikon D80 camera (Nikon Corporation, Tokyo, Japan) without pharmacological dilation of the pupil. AMD was categorized by independent graders using a modified Wisconsin protocol.^29^ Main features for each image were assessed with standardized photographs and included:

• Hard drusen size <63 μm, with more than ten hard drusen required to be present for the lesion to be classified as present;
• Soft drusen of size ≥125 μm, with presence of one soft drusen sufficient for classification of lesion to be present;
• Geographic atrophy;
• Choridal neovascularisation;
• Retinal Pigment Epithelium (RPE) detachment; and
• Disciform scar.

The prominent phenotype observed or the most severe lesion for each eye was used as the final grading for that eye. Individual categorization of AMD lesion was based on the more severely affected eye.

### Statistical analysis

The data was initially explored through descriptive analysis of variables using *t*-tests for quantitative variables and a χ^2^ test for categorical variables to compare different groups. Exploratory descriptive analysis was performed using quintiles of deprivation (IMD score). This initial exploration showed that there was a lower frequency of AMD in the most affluent areas, whereas similar proportions of AMD were detected in IMD quintiles 2–5. As the index is a relative rank rather than an absolute measure, the authors examined people living in the 5% most affluent areas to maximize power to detect an association between area deprivation and AMD.

Crude associations of both AMD and IMD measures with potential confounders were explored using univariable logistic regression, tabulation and χ^2^ tests. A stepwise logistic regression model was used to examine the effect of the highest fifth centile of deprivation (most affluent) on odds of AMD. Both education and social class were included in the final model to determine the relationship between area deprivation, individual SES and AMD. Furthermore, smoking was also included as an *a priori* confounder since it is a strong risk factor for AMD. Potential interactions between IMD and co-variables in logistic regression analyses were tested using interaction terms. All statistical analyses were conducted using STATA 12 (Statacorp, College Station, Texas, USA).

## Results

Of 8623 participants examined in the EPIC-Norfolk Eye Study, retinal photographs were obtained from 7501 (87.0%) participants of which 5344 pairs (62.0%) were of sufficient quality for grading of AMD related fundus lesions. Participants with missing or excluded photographs were older (70.8 years vs 67.4 years *P* < 0.01) and more likely to be male (47.5% of those missing vs 43.1% with gradable photographs *P* < 0.01), with lower levels of education (28.4% of missing with no formal qualifications vs 25.1% with graded photographs, *P* < 0.01). Similar proportions of smokers did not have gradable photos compared to those with grading. (8.61% vs 9.37% missing *P* = 0.23). The mean age of the 5344 included participants was 67.4 years (range 48.4–91.9 years), with 56.9% female. Women were younger than men, with a mean age of 66.9 years compared to 68.1 years for men (*P* < 0.01). A further 62 participants with missing exposure data were excluded, leaving 5182 included in the study.

Of the 5182 participants, AMD was identified in 653 participants (12.60%, 95% CI = 11.7–13.5%), of which 27 (0.52%, 95% CI = 0.34–0.76%) had evidence of late AMD and 626 (12.08%, 95% CI = 11.20–13.00%) early AMD. Of the 27 people with advanced AMD, 3 participants (11.1%) had severe visual impairment (VA<6/60), 4 (14.8%) had mild visual impairment (VA6/12-6/18) and 18 (66.7%) had good vision (VA>6/9.5).

A descriptive analysis using quintiles of deprivation is presented in Table 1. There was evidence of differences in the mean age of people living in different areas, with the oldest in the most deprived areas and the youngest living in relatively affluent areas (quintile 2), although the relationship was not linear. Although the lowest proportion of females lived in the least deprived areas, there was no statistical evidence of an association between sex and area deprivation. There was a strong association between education, social class, smoking, physical activity and area deprivation. There were higher proportions of people with lower educational attainment and from manual social classes in the most deprived areas. In addition, people living in the most affluent areas were more likely to be habitually active and never have smoked. There was no association between alcohol intake and quintiles of deprivation. People with AMD were more likely to live in more deprived areas, though there was no evidence of a trend across quintiles. Similarly, there were lower proportions of people with AMD living in the most affluent areas, again with no statistical evidence of a trend across quintiles.

The univariable associations with AMD are shown in Table 2. There was a crude association between the most affluent 5% and odds of AMD with some evidence of a protective effect, with people living in affluent areas being 37% less likely to have AMD (OR = 0.63, 95% CI = 0.40–0.98, *P* = 0.03). There was also a statistically significant association with sex and physical activity. Women had greater odds of AMD, whilst people reporting higher levels of physical activity were less likely to have AMD. People with A levels had lower odds of AMD compared to those without O levels (lowest level of educational attainment), though there was no evidence of a linear association with education overall. Smoking, alcohol and social class were not associated with AMD for the present study cohort.

In a multivariable analysis adjusting for age, sex, education, social class and smoking, people living in the most affluent areas had nearly half the odds of AMD compared to those living in comparatively more deprived areas (OR = 0.56, 95% CI = 0.36–0.89, *P* = 0.02) (Table 3). Physical activity was no longer associated with AMD after adjusting for age, indicating that older people were more likely to have both AMD and lower levels of physical activity.

## Discussion

The EPIC Norfolk Eye Study is the first study to examine the relationship between area deprivation and AMD. Age related macular degeneration is an important public health problem, with rising incidence and prevalence due to an ageing population,^2^ and no suitable treatment for a majority of cases.^30^ The adverse impact of sight loss extends beyond loss of visual function with increased risk of depression and falls leading to significant impact on health and social care. The authors have shown that people living in the most affluent areas have nearly half the odds of AMD compared to those living in more deprived areas, after adjusting for age, sex, education, social class and smoking. These findings support those of other studies relating to AMD and individual SES, including findings from the Singapore Malay eye study, which found a two-fold increase in AMD amongst people with the lowest levels of educational attainment.^31^ The findings contrast to those of several other studies that have failed to detect an association between AMD and education, including results from India^32^ and the United States.^11,33^ Although Zhang and co-workers showed that educational attainment was not significantly associated with AMD in the National Health and Nutrition Examination Surveys (NHANES III and NHANES 2005–8), they found higher age- and sex-standardized prevalence of AMD in people with lowest levels of income compared to those with the highest income level in NHANES III (17.9 vs 11.5% *P* = 0.03), but not in NHANES 2005–8 (10.4% vs 6.8%, *P* = 0.06).^11^

The authors have found that people living in the most affluent areas of Norfolk had reduced odds of AMD, using the English IMD measure. IMD is a continuous measure of relative deprivation and does not have a predetermined cut off to identify those who are deprived, rather, cut-offs are determined by users to serve the purpose of their analysis.^28^ It is common to examine area deprivation from the most deprived perspective, using the lowest 5th or 10th centiles as an arbitrary threshold. However, there was no association found when using the most deprived 5% as a cut off in this population based study. This was due to a relatively affluent population overall in this study. All participants in the most affluent quintile of the study population IMD rankings were also in the most affluent quintile using the national IMD distribution. On the other hand, only 16% of study participants living in the most deprived quintiles locally would have met the cut off for the most deprived quintile nationally. Overall, only 3% of the 5182 participants were in the most deprived quintile using national IMD rankings. There are two inferences from this lack of effect in the most deprived groups from this study. Firstly, any increased risk in the most deprived populations may have resulted from poverty – that is, a threshold effect may exist that was not detected in the studied population since there were insufficient numbers of participants who lived in the poorest conditions to have met this threshold. Secondly, following on, the present study did not have statistical power to detect an effect in the most deprived populations. There was no statistical evidence of a linear or gradient effect of IMD on AMD prevalence, with similar proportions of AMD in IMD quintiles 2–5. Yet a threshold effect was found at the other end of the scale, with reduced odds of AMD in the most affluent people. The contrasting results are difficult to explain, but may be due to a partially observed gradient effect, chance or selection bias, as discussed below.

The present study also showed that the association between area deprivation and AMD was independent of individual SES, age, sex and smoking. Smoking is a strong risk factor for AMD^34^ and there is a higher prevalence of smokers in deprived areas.^35^ Therefore, smoking is a strong candidate for mediating the effect of deprivation on AMD. However, in this population based study, despite people living in more deprived areas being more likely to be current smokers, no association was detected between cigarette smoking and AMD, likely due to low statistical power in a healthy population with a low prevalence of current smokers. In the present cross sectional study, only 4.3% of participants were current smokers. During the first health check of EPIC-Norfolk between 1993 and 1997, 8.6% of participants were current smokers, which was substantially lower than 18.5% reported in the pooled analysis of baseline data from the Beaver Dam, Blue Mountains and Rotterdam cohort studies, which also examined predominantly Caucasian populations.^34^

Therefore although these results indicated that the association between IMD and AMD was independent of smoking, this mechanism cannot be ruled out in other populations. Other potential downstream mechanisms of deprivation include lifestyle risk factors such as diet, since people living in more deprived neighbourhoods have lower levels of serum carotenoids,^36^ and lutein and zeaxanthine are principle components of macular pigment with important roles in visual function.^37^ In addition to increased risk of AMD aetiology, people living in deprived areas may also have worse outcomes due to poor access to health care; this can be mediated through lower levels of health knowledge and therefore less frequent access to routine preventive eye examinations with optometrists. The authors have previously demonstrated an association between IMD and low vision, which was partly mediated through uncorrected refractive error, indicating access to optometry and primary eye care could play a role in this association.^38^ However, there was no evidence of higher proportions of people with uncorrected refractive error (URE) and AMD in the present study (of people with AMD: 13.50% with URE vs 12.46% without; analysis not shown).

The EPIC-Norfolk Eye Study was nested within the third follow-up of a longitudinal study and results are affected by a relatively healthy selected population. Healthy survivors are less likely to have risk factors and disease and this is demonstrated by a low prevalence of AMD and smokers in this study. Furthermore, people with poor health and visual function are less likely to attend for a clinical examination, thus exacerbating the healthy survivor effect. The impact of this limitation may have been reduced statistical power of the study to detect an association, in particular between smoking and AMD. The EPIC-Norfolk population was also relatively affluent, which would have reduced the prevalence of disease further. However, the impact of these limitations cannot be quantified and may have resulted in an under or over estimate of the main relationship examined. The relatively healthy and affluent population also limits the generalizability of the study results, since it is not representative of the general UK population. However, although point estimates may differ, the underlying relative associations may be observed in other populations. The limitations of the present study indicate that the results should be interpreted with caution and further studies of area deprivation and AMD are recommended.

The EPIC-Norfolk Eye Study is the first to demonstrate an association between area deprivation and AMD, independent of individual SES. The authors have also shown that this effect was independent of smoking, and requires further investigation in other populations.

## Author statements

### Acknowledgements

We would like to thank Mr Pak S. Lee for the training of research clinic nursing staff and equipment maintenance, Nichola Dalzell for running the health examination clinic and all the EPIC-Norfolk participants for their time and support.

### Ethical approval

The study was approved by the Norfolk Local Research Ethics Committee and adhered to the Declaration of Helsinki. All participants gave written informed consent.

### Funding

EPIC-Norfolk infrastructure and core functions are supported by grants from the Medical Research Council (G0401527) and Cancer Research UK (C864/A8257). The clinic for the third health examination was funded by an Age UK Research into Ageing grant (262). The pilot phase was supported by Medical Research Council (G9502233) and Cancer Research UK (C864/A2883). Dr Yip is a National Institute for Health Research (NIHR) Clinical Lecturer. Dr Khawaja is a Wellcome Trust Clinical Research Fellow. Michelle Chan is an MRC/RCOphth Clinical Training Felllow and has received additional support from the International Glaucoma Association. Professor Foster has received additional support from the Richard Desmond Charitable Trust (via Fight for Sight). Professor Foster and Tunde Peto received funding from the Department for Health through the award made by the NIHR to Moorfields Eye Hospital and the UCL Institute of Ophthalmology for a specialist Biomedical Research Centre for Ophthalmology. None of the funding organizations had a role in the design or conduct of the research.

### Conflict of interest

The authors declare no conflict of interest.

## Figures and Tables

**Table 1:** Descriptive analysis of 5182 participants from the EPIC Norfolk Eye study by quintile of index of multiple deprivation.

	Quintile 1 (least deprived)		Quintile 2		Quintile 3		Quintile 4		Quintile 5 (most deprived)		*P*-value*
Age (Years)	67.6	(7.5)	66.8	(8.1)	67.2	(7.7)	67.5	(7.7)	67.9	(7.2)	0.02
Sex											0.48
Male	475	(45.6)	436	(41.9)	428	(42.7)	463	(43.9)	445	(42.6)	
Female	567	(54.4)	605	(58.1)	575	(57.3)	592	(56.1)	596	(57.3)	
Education											<0.01
Less than O level	161	(12.5)	267	(20.8)	249	(19.4)	288	(22.4)	319	(24.8)	
O levels	118	(18.5)	139	(21.8)	130	(20.4)	130	(20.4)	121	(19.0)	
A levels	496	(21.3)	481	(20.6)	447	(19.2)	471	(20.2)	438	(18.8)	
Degree or higher	267	(28.8)	154	(16.6)	177	(19.1)	166	(17.9)	163	(17.6)	
Social class											<0.01
Non-manual	844	(24.7)	650	(19.0)	657	(19.2)	657	(19.2)	610	(17.9)	
Manual	198	(11.2)	391	(22.2)	346	(19.6)	398	(22.6)	431	(24.4)	
Smoking											<0.01
Current	32	(14.4)	44	(19.8)	31	(14.0)	48	(21.6)	67	(30.2)	
Former	448	(19.3)	441	(19.0)	454	(19.5)	472	(20.3)	507	(21.8)	
Never	562	(21.3)	556	(21.1)	518	(19.6)	535	(20.3)	467	(17.7)	
Physical activity											<0.01
Inactive	313	(17.2)	352	(19.4)	360	(19.8)	396	(21.8)	398	(21.9)	
Moderately inactive	364	(23.3)	311	(20.0)	309	(19.8)	291	(18.7)	285	(18.3)	
Moderately active	182	(19.2)	211	(22.3)	174	(18.4)	192	(20.3)	189	(20.0)	
Active	183	(21.4)	167	(19.5)	160	(18.7)	176	(20.6)	169	(19.8)	
Alcohol intake (Units in previous week)	8.1	(9.0)	7.1	(9.0)	7.6	(8.6)	7.6	(9.8)	7.5	(9.6)	0.25
Visual impairment	30	(20.7)	26	(17.9)	23	(15.9)	33	(22.8)	33	(22.8)	0.69
AMD	123	(18.8)	136	(20.8)	129	(19.8)	131	(20.1)	134	(20.5)	0.92

Data presented as *n*(%) for categorical and mean (SD) for quantitative variables. *P-value from χ^2^ test for trend for categorical and analysis of variance for continuous variables.

**Table 2:** Univariable associations between sociodemographic, lifestyle risk factors and AMD.

	OR	(95% CI)	*P*-value*
Age (Per year increase)	1.07	(1.06–1.08)	<0.01
Sex			
Male	Ref		
Female	1.26	(1.07–1.49)	<0.01
Education			0.07^a^
Less than O level	Ref		
O levels	0.78	(0.59–1.04)	0.09
A levels	0.77	(0.63–0.94)	<0.01
Degree or higher	0.82	(0.64–1.05)	0.12
Social class			0.98
Non-manual	Ref		
Manual	1.00	(0.84–1.19)	0.98
Smoking			0.32^a^
Current	Ref		
Former	1.39	(0.87–2.21)	0.17
Never	1.41	(0.88–2.24)	0.15
Physical activity			<0.01^a^
Inactive	Ref		
Moderately inactive	0.78	(0.64–0.96)	0.02
Moderately Active	0.76	(0.60–0.97)	0.03
Active	0.64	(0.50–0.83)	<0.01
Alcohol intake (Per units in previous week)	1.00	(0.99–1.01)	0.46
IMD (most affluent 5%)	0.63	(0.40–0.98)	0.03

OR=Odds ratio, AMD = age related macular degeneration. *P-value from Wald test for association.

a Likelihood ratio test for significance of categorical variables.

**Table 3:** Multivariable associations between area deprivation, sociodemographic, lifestyle risk factors and AMD.

	OR	(95% CI)	*P*-value*
Age (years)	1.07	(1.06–1.09)	<0.01
Sex			
Male	Ref		
Female	1.39	(1.16–1.66)	<0.01
Education			0.48^a^
Less than O level	Ref		
O levels	0.99	(0.74–1.34)	0.97
A levels	0.94	(0.76–1.16)	0.58
Degree or higher	1.14	(0.87–1.51)	0.34
Social class			
Non-manual	Ref		
Manual	1.03	(0.85–1.24)	0.78
Smoking			0.94^a^
Current	Ref		
Former	1.06	(0.66–1.71)	0.81
Never	1.08	(0.67–1.74)	0.75
IMD (Most affluent 5%)	0.56	(0.36–0.89)	0.02

*P-value from Wald test for quantitative, binary and individual levels of categorical variables; from. AMD = age related macular degeneration IMD = index of multiple deprivation.

a Likelihood ratio test for significance of categorical variables.
